# Making head or tail of cnidarian *hox* gene function

**DOI:** 10.1038/s41467-018-04585-y

**Published:** 2018-06-05

**Authors:** Fabian Rentzsch, Thomas W. Holstein

**Affiliations:** 1 Sars Centre for Marine Molecular Biology, University of Bergen, Thormøhlensgt 55, 5008 Bergen, Norway; 2 Molecular Evolution & Genomics, Centre for Organismal Studies Heidelberg, Heidelberg University, Im Neuenheimer Feld 230, 69120 Heidelberg, Germany

## Abstract

Distantly related animals have spectacularly different shapes and body plans, which can render it difficult to understand which of their body parts may have a shared evolutionary origin. Studying the molecular regulation of the development of these body parts during embryogenesis can help identifying commonalities that are not visible by eye.

## Abstract (web summary)

*Hox* genes are known to regulate head-tail axis patterning but their molecular role in animals diverged from the bilaterian lineage is unclear. Here, Fabian Rentzsch and Thomas W. Holstein comment on a paper by Mark Martindale and colleagues on the functional role of two *Hox* genes in axial patterning of the sea anemone.

## Introduction

A new study employs this approach to address a fundamental question, the origin of the head-to-tail axis of bilaterians. The results show that two *Hox* genes—key regulators of the patterning of this axis in bilaterians—have important roles in axial patterning in a sea anemone, which belongs to a group of animals that diverged from the bilaterian lineage more than 600 million years ago. Here, we discuss the implications of these interesting new findings in the light of previous observations on axial patterning in bilaterian and non-bilaterian animals.

## Axial patterning in bilaterians and cnidarians

**Fig. 1:**
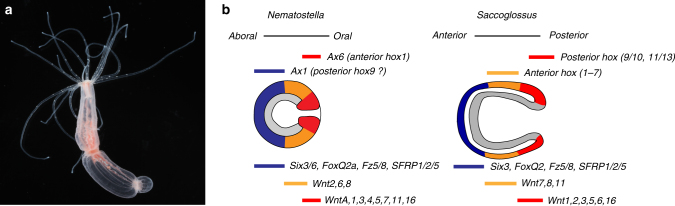
*Nematostella* and the evolution of axial patterning systems. **a** A photograph of an adult *Nematostella vectensis* polyp. A ring of tentacles that are used for capturing prey surrounds the oral opening (oriented to the top). The “oral” opening serves as both mouth and anus and derives directly from the blastopore (to the right in **b**). **b** Schematic drawing of gastrula stages of *Nematostella vectensis* and the hemichordate *Saccoglossus kowalevskii*, which serves as an example for a bilaterian embryo. The blastopore is oriented to the right. Relative gene expression domains are indicated to illustrate the support for different scenarios in the evolutionary relation of the primary body axis. Note that the gene expression domains are simplified and that some of the *Nematostella* Wnts are expressed in the endoderm. Data for *Nematostella* are summarized in refs. ^5,12,13^, and for *Saccoglossus* in ref. ^15^. Photo credit for **a**: Chiara Sinigaglia

The body plan of bilaterians (e.g. vertebrates, arthropods and many worm-shaped animals) can be described by a Cartesian coordinate system with three body axes^1^: the anterior–posterior (AP) axis that is oriented parallel to the gut, the dorsal–ventral (DV) axis defining the “back” and “belly” sides and the left–right (LR) axis for laterality. Increasing evidence indicates that the molecular basis of the formation and patterning of these axes is highly conserved within bilaterians, suggesting that at least the AP and DV axes were present already in the last common ancestor of bilaterians. For the AP axis, Wnt/β-Catenin signalling determines the site of gastrulation and endomesoderm formation, and it suppresses anterior and promotes posterior ectodermal development^2^. Interesting and longstanding questions are the relation of the bilaterian body axes to those of pre-bilaterians and the transition from radial to bilateral symmetry in animal evolution^3,4^. Here, cnidarians (i.e. jellyfish, hydroids, corals and sea anemones) are of critical importance: they represent the well-established sister group to all bilaterian animals and they exhibit a gastrula-like body plan with a primary radial symmetry^5^ (Fig. 1a). Morphological characters that identify the orientation of the AP axis in most bilaterians (i.e. an anterior brain-like centralization of the nervous system and a through gut with the mouth located anterior to the anus) are not present in cnidarians. A key question is, therefore, which of the molecular vectors (i.e. signalling networks and transcription factors) defining the bilaterian body axes are acting in cnidarian axis formation.

## Function of cnidarian *Hox* genes

In an interesting and important recent paper, DuBuc et al.^6^ have addressed the role of two *Hox* genes in axis formation and oral/aboral markers in the sea anemone *Nematostella vectensis*, which is a major cnidarian model system^5^. Hox transcription factors have a conserved role in the patterning of the bilaterian AP axis^7^. There are three groups of bilaterian *Hox* genes: anterior, central and posterior ones. They have sharp anterior borders of expression, whereas the posterior limit of their expression is often less clearly defined. The position of the anterior expression border differs for different groups of *Hox* genes: those of anterior *Hox* genes are located more anteriorly than those of central and posterior *Hox* genes. The picture emerging from functional studies (mainly in arthropods and vertebrates) is that *Hox* genes act from gastrulation stages onwards to provide specific identities to consecutive territories along the AP axis, with the exception of the anterior-most region, which is typically free of *Hox* gene expression. While *Hox* genes are essential for the patterning of large parts of the AP axis, they have no role in the initial establishment of the body axis^7^. Cnidarian *Hox* genes have been identified 26 years ago^8^ and reveal a broad spectrum of expression patterns, but only now the first functional analysis of *Hox* genes during cnidarian embryonic development has been presented by DuBuc et al.^6^. Cnidarians have anterior, but no central, *Hox* genes and they have genes that have been classified as posterior in some studies and as unresolved non-anterior genes in others^5,9,10^. DuBuc et al. focus on one anterior (*NvAx6*) and one putative posterior (*NvAx1*) *Hox* gene in *Nematostella*^6^.

In situ hybridization analyses show opposing expression patterns of *NvAx1* and *NvAx6*. *NvAx1* is expressed in blastulae at the presumptive aboral side and Nv*Ax6* at the presumptive oral side. In *NvAx1* knockdown animals, the expression of aboral-most genes is lost, whereas broader aboral markers (*NvSix3/6*) and oral markers are not or only slightly affected. Upon knockdown of *NvAx6*, the animals fail to complete gastrulation, and the expression of oral markers, including four *Wnt* genes, is lost. The domain of *NvWnt2* (the most aboral cnidarian *Wnt* gene) is shifted towards the oral pole, but still present. Since knockdown of *NvAx1* and *NvAx6* results in an expansion of *NvAx6* and *NvAx1*, respectively, the authors tested by double knockdown whether the expanded expression contributes to the observed phenotypes. After double knockdown, the animals gastrulate, form an endodermal tissue layer and display normal expression of oral genes at gastrula stage (although not at later stages). Unexpectedly, however, the expression of the aboral gene *NvSix3/6* is lost in double knockdowns at the gastrula stage, even though single knockdowns show a mild reduction (*NvAx1*) and expansion (*NvAx6*), respectively. In overexpression experiments, injection of *NvAx1* mRNA suppresses *NvAx6* expression, whereas *NvAx6* mRNA injection does not suppress *NvAx1*. These experiments clearly show that *NvAx1* is required for aboral and *NvAx6* for oral development in *Nematostella* and that they mutually inhibit each other’s expression.

The relation between Wnt signalling and *NvAx6* function remains to be determined, since the earliest analyses of the functional perturbations of the *Nematostella Hox* genes are performed at the gastrula stage, after the patterning of the oral domain at the blastula stage^11^. Nevertheless, the new data unambiguously show that *Hox* genes function in the development of the oral–aboral axis in *Nematostella*.

## *Hox* genes and the evolutionary origin of the bilaterian AP axis

What does this mean for the evolutionary relation of the oral–aboral axis of cnidarians to the AP axis of bilaterians? The authors suggest that the roles of a *Nematostella* anterior *Hox* gene in oral, and a putative posterior *Hox* gene in aboral development support homology of the cnidarian oral to bilaterian anterior, and aboral to posterior domains. Challenging this hypothesis requires a closer look at the similarity to the role of *Hox* genes in bilaterians, and, importantly, consideration of existing data on axial patterning in *Nematostella* and other cnidarians.

*NvAx1* and *NvAx6* appear to function during the blastula stage and they are required for oral-most (*NvAx6*) and aboral-most (*NvAx1*) development, while the identity in the domains in between the two poles (based on gene expression, e.g. *NvWnt2*) is unchanged, though displaced towards the oral pole. This is different from the conserved role of bilaterian *Hox* genes, which act during and after gastrulation, confer a molecular identity to consecutive areas and have no role in anterior-most development. Despite these differences, it is not inconceivable that the function of the *Hox* system in cnidarians (as described by DuBuc et al.^6^) and in bilaterians (in AP patterning) had a shared evolutionary origin.

## Wnt signalling and the evolutionary origin of the bilaterian AP axis

The hypothesis devised by DuBuc et al.^6^ is, however, contradicted by several publications on the molecular control of axial patterning in *Nematostella* and other cnidarians. In *Nematostella*, *Wnt* genes are expressed exclusively in the oral domain from blastula stages on and β-catenin is nuclearized predominantly in the oral area^4,5,12^. Wnt signalling is required for endomesoderm development and the genes expressed in the oral domain are most sensitive to reductions of Wnt/β-catenin signalling at the blastula stage^5,12,13^. These Wnt functions in oral development are strikingly similar to those in posterior development of bilaterians and well conserved in other cnidarians^2^. In contrast, development of the aboral domain requires protection from high levels of Wnt/β-catenin signalling. The transcription factors *NvSix3/6* and *NvFoxQ2* and the Wnt modulators *NvSFRP1* and *NvFz5/8* are expressed in the anterior domain of nearly all bilaterians that have been studied and their *Nematostella* orthologs are expressed in the aboral domain^5,13^. The establishment of this domain at blastula stage requires low level Wnt/β-catenin signalling and MAPK activity^13,14^. Subsequently, *NvSix3/6* and, to a lesser extent, *NvFoxQ2a* control the further development of the domain, whereas *NvFz5/8* prevents the expansion of the aboral domain towards more oral regions^5,13^.

These data support the hypothesis that with respect to the molecular regulation of axial patterning, the aboral domain of cnidarians is homologous to the anterior domain in bilaterians, and the oral domain to the posterior domain (Fig. 1b). This would mean that the roles of *NvAx1* and *NvAx6* in axial patterning evolved independently of those of *Hox* genes in bilaterians.

## Perspective

The remarkable and uncommon early roles of *Hox* genes in *Nematostella* identified by DuBuc et al. highlight, however, that the axial patterning systems of cnidarians and bilaterians (and within the two groups) are not simply copies of each other, but rather have undergone substantial modifications during their independent evolution. In future studies, appreciating both conserved and divergent aspects of axial patterning mechanisms will certainly benefit the understanding of their evolution. With respect to Wnt signalling and the *Hox* genes, it will be interesting to address the mutual effects of manipulating Wnt signalling and *NvAx6* at earlier blastula stages, when patterning of the oral–aboral axis commences. Other important questions are the molecular mechanism that localizes Wnt/β-catenin signalling components to the animal domain in *Nematostella*, but to the vegetal pole in bilaterians^4^^,^^5^, and the function of other *Nematostella Hox* genes, whose staggered expression along the secondary body axis resembles bilaterian-type *Hox* expression more than that of *NvAx1* and *NvAx6*^4,10^. Undoubtedly, the work by DuBuc et al. will serve as an important reference for any new studies on axial patterning in cnidarians, which may ultimately help us in understanding the evolutionary origin of bilaterally symmetric body plans.
